# Non-Pharmacological Mitigation of Acute Mental Stress-Induced Sympathetic Arousal: Comparison Between Median Nerve Stimulation and Auricular Vagus Nerve Stimulation

**DOI:** 10.3390/s25051371

**Published:** 2025-02-23

**Authors:** Yuanyuan Zhou, Sina Masoumi Shahrbabak, Rayan Bahrami, Farhan N. Rahman, Jesus Antonio Sanchez-Perez, Asim H. Gazi, Omer T. Inan, Jin-Oh Hahn

**Affiliations:** 1 Mechanical Engineering, University of Maryland, College Park, MD 20742, USA; yzhou114@umd.edu (Y.Z.); smasoumi@umd.edu (S.M.S.); rayan@umd.edu (R.B.); 2 Electrical and Computer Engineering, Georgia Institute of Technology, Atlanta, GA 30332, USA; farhan.rahman@gatech.edu (F.N.R.); omer.inan@ece.gatech.edu (O.T.I.); 3 Electrical and Computer Engineering, University of Puerto Rico at Mayagüez, Mayagüez, PR 00680, USA; jesus.sanchez25@upr.edu; 4 Computer Science, Harvard University, Allston, MA 02134, USA; agazi@g.harvard.edu

**Keywords:** acute mental stress, peripheral neuromodulation, median nerve stimulation, auricular vagus nerve stimulation

## Abstract

Acute mental stress is a common experience in daily life, significantly affecting both physiological and psychological well-being. While traditional pharmacological interventions can be effective, they often accompany undesirable side effects. Non-pharmacological alternatives, such as non-invasive transcutaneous peripheral neuromodulation, have promise in mitigating acute stress-induced arousal, possibly with fewer side effects. Median nerve stimulation (MNS) and auricular vagus nerve stimulation (AVNS), in particular, have demonstrated notable potential. However, efficacy and mechanism of action pertaining to MNS and AVNS remain largely unknown. This paper comparatively investigated MNS and AVNS in terms of efficacy and mechanism of action in the context of mitigating acute stress-induced arousal. Using an experimental dataset collected from 19 healthy participants who experienced acute mental stressors as well as MNS and AVNS, we showed that (i) MNS and AVNS are both effective in mitigating acute stress-induced cardiovascular arousal with MNS modestly superior to AVNS in terms of a synthetic multi-modal variable derived from physio-markers representing heart rate, blood pressure, stroke volume, cardiac output, and peripheral vasoconstriction: 74% vs. 71% in explainability; 86% vs. 69% in stimulation consistency; 0.77 vs. 0.40 in stimulation sensitivity; and 34% vs. 19% in stimulation effectiveness, respectively; and that (ii) MNS and AVNS mitigate acute stress-induced cardiovascular arousal in a distinct mechanism of action: MNS primarily mitigates the arousal pertaining to the physio-markers representing heart rate and peripheral vasoconstriction, while AVNS mitigates the arousal pertaining to the physio-markers representing heart rate, blood pressure, stroke volume, cardiac output, and peripheral vasoconstriction. These findings may help to support future device development for addressing acute mental stress-induced arousal through MNS or AVNS, and they pave the way toward a better understanding of how to quantify the efficacy of such interventions.

## 1. Introduction

Acute mental stress is prevalent in daily life, with excess stress exerting negative impacts on both physiological and psychological well-being. Acute stress plays a significant role in the onset and progression of various health and disease conditions, including cardiovascular diseases, anxiety disorders, and immune dysfunction [1,2,3,4,5,6,7,8]. While conventional treatment approaches based on pharmacological interventions can be effective in managing chronic stress (at a timescale of days and months), they are not suited to addressing acute stress episodes (at a timescale of seconds). Additionally, these interventions often accompany undesirable side effects and have the risk of dependency [9,10,11,12]. As a result, there has been growing interest in non-pharmacological alternatives, which include cognitive behavioral therapy, mindfulness-based interventions, bio-feedback, physical exercise, and non-invasive peripheral neuromodulation—electrical stimulation of peripheral nerves with the aim of providing afferent feedback to the brain to reduce sympathetic arousal induced by acute stress—to list a few [12,13,14,15,16,17,18,19,20,21,22,23,24,25,26,27,28,29,30]. In particular, non-invasive peripheral neuromodulation has emerged as an outstanding breakthrough for mitigating acute stress-induced arousal by improving emotional regulation and reducing limbic brain activity, thereby promoting relaxation and enhancing mental well-being [19,20,21,22,23,24,25,26,27,28,29,30].

Non-invasive peripheral neuromodulation modulates certain aspects of physiological processes by applying stimulation (in the form of, e.g., electrical, magnetic, or vibrational) to target peripheral nerves (particularly those associated with the regulation of acute stress-induced arousal). This approach has gained significant attention by virtue of its ability to reduce stress-induced arousal by decreasing sympathetic activity and enhancing parasympathetic activity. Several stimulation sites have been explored, including the auricular (ear) branch of the vagus nerve [25,26,28], cervical vagus nerve [19,24,25,27], and median nerve [25,29,30] to list a few. While many of these sites show potential for mitigating stress-induced arousal, certain stimulation sites appear to offer more targeted and effective benefits than other sites.

Among various neuromodulation techniques, median nerve stimulation (MNS) and auricular vagus nerve stimulation (AVNS) possess unique advantages in mitigating acute stress-induced arousal. First, both MNS and AVNS can provide practical solutions to mitigate acute stress-induced arousal in real time using wearables. MNS can be provided at the wrist, potentially using a wristband, and AVNS can be provided using hearables. Second, both MNS and AVNS can regulate acute stress-induced physiological responses via their connection to the autonomic nervous system. In fact, MNS [25,29,30] and AVNS [25,26,28] result in a reduction in sympathetic activity. Notably, we demonstrated that MNS and AVNS were effective in reducing stress-induced sympathetic arousal relative to sham stimulation [25]. For these reasons, both MNS and AVNS stand out as attractive stimulation modalities by virtue of their practical and efficacious merits.

Despite the aforementioned advantages, a critical knowledge gap remains regarding the relative efficacy and mechanism of action pertaining to various peripheral neuromodulation modalities, including MNS and AVNS. Which peripheral neuromodulation modality is the most efficacious in mitigating acute stress-induced arousal remains unknown. Further, how each peripheral neuromodulation modality mitigates acute stress-induced sympathetic arousal remains poorly understood.

In our initial attempt to address this knowledge gap, we comparatively investigated MNS and AVNS in terms of efficacy and mechanism of action in the context of mitigating acute stress-induced arousal. Using an experimental dataset collected from 19 healthy participants who experienced acute mental stressors while receiving MNS and AVNS, we analyzed the responses of physio-markers representing heart rate, blood pressure, stroke volume, cardiac output, and peripheral vasoconstriction as well as a synthetic multi-modal variable (SMV) developed in our prior work [31] to acute stressors as well as MNS and AVNS.

This paper is organized as follows. Section 2 presents the experimental dataset, physiological signal processing and analysis procedure, and our approach to compare MNS and AVNS in terms of efficacy and mechanism of action. Section 3 presents key results. Section 4 discusses the results and explains the implications of the results in the context of efficacy and mechanism of action pertaining to MNS and AVNS. Section 5 concludes the paper by summarizing the main contributions and suggesting potential directions for future research.

## 2. Methods

### 2.1. Experimental Dataset and Protocol

**Figure 1:**
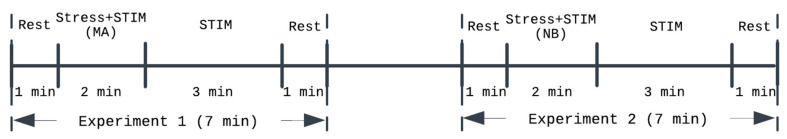
Experimental protocol. MA: mental arithmetic test. NB: N-back test. STIM: stimulation (MNS or AVNS).

We used an experimental dataset collected from 19 young healthy participants with no known history of neuropsychiatric disorder (sex: 9 male and 10 female; age: 21 ± 2, height: 169 ± 11 cm; weight: 64 ± 12 kg), which is part of a larger dataset we collected under the approval of the Institutional Review Board at Georgia Institute of Technology (H18452) and the Navy Human Research Protection Office [25]. In brief, each participant completed the study protocol outlined in Figure 1 twice in two separate visits. The protocol included two experiments, each of which was composed of (i) an initial rest period, a 1 min period during which baseline physiological state was measured; (ii) administration of an acute mental stressor and stimulation (“Stress + STIM” in Figure 1); (iii) administration of stimulation alone (“STIM” in Figure 1); and (iv) final rest period, a 1 min period during which physiological deviations returned to near the baseline. In random order, we used MNS in one visit and AVNS in the other visit. In each visit, we used a mental arithmetic test in the first experiment (“Experiment 1” in Figure 1) and the N-back test in the second experiment (“Experiment 2” in Figure 1) as acute mental stressors.

We used a DS8R current stimulator (Digitimer, Broadway, Letchworth Garden City, UK) to administer MNS (on the left wrist) and AVNS (on the left ear) by delivering a pulsed 1-cycle square alternating symmetrical bi-phasic waveform with a fixed inter-phase interval of 1 µs. For MNS, we used a pulse width of 200 μs and a trigger duration of 100 ms according to a prior work [32]. For AVNS, we used a pulse width of 500 μs and a trigger duration of 40 ms likewise according to a prior work [33]. For both MNS and AVNS, we determined the maximum tolerable stimulation intensity on a participant-by-participant basis and used its 66.7% level as the stimulation intensity in the experiments.

In this work, we used 3 physiological signals continuously recorded during these experiments at a 2 kHz sampling rate: the electrocardiogram (ECG), the photoplethysmogram (PPG), and the seismocardiogram (SCG). We employed a BN-RSPEC (Biopac Systems, Goleta, CA, USA) with surface electrodes to record the ECG, a BST09001S (Shanghai Berry Electronic Tech, Shanghai, China) placed at a fingertip interface with a PPG100C (Biopac Systems, Goleta, CA, USA) to record the PPG, and a custom-designed accelerometer package placed on the chest to record the SCG. We synchronously recorded these physiological signals using an MP150 and its accompanying Acqknowledge software (Biopac Systems, Goleta, CA, USA).

### 2.2. Data Processing

**Figure 2:**
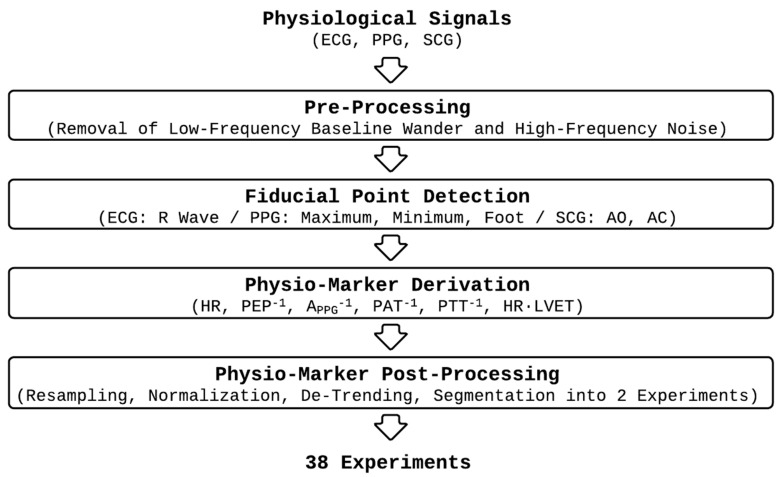
Data processing procedure.

We processed the experimental dataset on a participant-by-participant basis as shown in Figure 2. Details follow.

First, we pre-processed the physiological signals to remove low-frequency baseline wander and high-frequency noise using the pipeline developed in our prior work [25,31,34,35,36,37].

Second, we detected fiducial points in the physiological signals required to derive physio-markers on a beat-by-beat basis as follows:

(1) We detected the R waves in the ECG as described in our prior work [25]. Then, we gated the PPG and the SCG signals into individual beats using the R waves as described in our prior work [25].
(2) From each PPG beat, we detected its maximum and minimum points as described in our prior work [25]. In addition, we detected its foot using the intersecting tangent method [38].
(3) From each SCG beat, we detected its AO and AC points [39] in a semi-automated fashion as described in our prior work [25].

Third, we derived 6 physio-markers on a beat-by-beat basis as follows, which were demonstrated in our prior work to be responsive to acute mental stressors:

(1) Heart rate (HR) as 60 divided by the instantaneous heart period (in seconds, derived as the time interval between the R waves in the ECG pertaining to current and next beats).
(2) Pre-ejection period (PEP) as the time interval between the R wave in the ECG and the AO point in the SCG.
(3) PPG amplitude (A_PPG_) as the amplitude of the PPG beat.
(4) Pulse arrival time (PAT) as the time interval between the R wave in the ECG and the foot of the PPG.
(5) Pulse transit time (PTT) as the time interval between the AO point in the SCG and the foot of the PPG.
(6) Left ventricular ejection time (LVET) as the time interval between the AO point and the AC point in the SCG.

Note that the above physio-markers are associated with physiologically plausible interpretations. First, HR tends to increase in response to acute mental stressors [34,40]. Second, PEP tends to be inversely proportional to cardiac contractility, and thus, stroke volume (SV) [41,42]. Given that SV tends to increase in response to acute mental stressors [34,43], PEP tends to decrease in response to acute mental stressors. Third, A_PPG_ at an extremity tends to be inversely proportional to the degree of peripheral vasoconstriction, and thus, total peripheral resistance (TPR) [44,45]. Given that peripheral vasoconstriction and TPR tend to increase in response to acute mental stressors [34], A_PPG_ tends to decrease in response to acute mental stressors. Fourth, PAT and PTT tend to be inversely proportional to systolic and diastolic blood pressures (BPs) [34]. Given that BP tends to increase in response to acute mental stressors [34,46], PAT and PTT tend to decrease in response to acute mental stressors. Fifth, LVET tends to be proportional to SV [41,47]. Hence, LVET tends to increase in response to acute mental stressors [34].

We defined the 6 physio-markers to standardize their direction of change in response to acute mental stressors and stimulation: HR, PEP^−1^, A_PPG_^−1^, PAT^−1^, PTT^−1^, and LVET. In this way, all the physio-markers tend to increase in response to acute mental stressors and decrease in response to MNS and AVNS. In addition, we normalized LVET by HR (i.e., HR·LVET) to compensate for its dependence on HR (namely, LVET decreases as HR increases).

Fourth, we post-processed the physio-markers on a participant-by-participant basis, as follows, to render the subsequent analysis more robust against inter- and intra-participant variability:

(1) We resampled the physio-markers at a 1 Hz sampling rate and then smoothed them using a 30 s moving-average filter.
(2) We normalized each physio-marker time series using the z-score normalization.
(3) We de-trended each physio-marker time series to remove drifts in each experiment by (i) calculating the average physio-marker values pertaining to the initial and final rest periods and (ii) subtracting the linear interpolation of these average physio-marker values from the experiment period. In this way, each experiment was de-trended separately.
(4) We segmented the physio-marker time series into the two experiments (“Experiment 1” and “Experiment 2”).

In sum, we obtained 38 experiments pertaining to both MNS and AVNS from the dataset of 19 participants, which were used in the subsequent analysis described in Section 2.3 and Section 2.4.

### 2.3. Construction of Synthetic Multi-Modal Variable (SMV)

Using the physio-markers obtained in Section 2.2, we constructed SMVs pertaining to MNS and AVNS as a measure of aggregated cardiovascular arousal in response to acute mental stressors as well as MNS and AVNS [31]. In brief, we constructed the SMV pertaining to an experiment as a linear combination of the physio-markers:

(1) $$\phi \left(y\right)=\sum_{i=1}^{N_{y}}\alpha_{i}y_{i}$$

where $\phi \left(y\right)$ is the SMV, y= {HR, PEP^−1^, A_PPG_^−1^, PAT^−1^, PTT^−1^, HR·LVET}, $\alpha_{i}$, $i=1,\cdots ,N_{y}$ are the weights pertaining to individual physio-markers in the SMV, and $N_{y}=6$ is the number of physio-markers. We determined the weights in the SMV (namely, $\alpha_{i}$’s) in the leave-one-experiment-out analysis setting as follows. For an experiment, we determined the weights in the SMV in Equation (1) using the physio-marker time series in the remaining 37 experiments, by solving a constrained optimization problem that promotes (i) zero SMV response in the rest period, (ii) positive SMV response to acute mental stressors, and (iii) negative SMV response to MNS and AVNS [31]. Then, we used Equation (1) to construct the SMV pertaining to the experiment based on the weights thus determined. We repeated the above procedure to construct distinct SMVs pertaining to MNS and AVNS in order to compare and contrast their respective mechanism of action.

### 2.4. Comparison Between MNS vs. AVNS

To comparatively investigate MNS and AVNS in terms of efficacy and mechanism of action in the context of mitigating acute stress-induced arousal, we devised and used the following metrics applicable to both SMV and individual physio-markers (note that a subset of these metrics was shown to be useful for the efficacy of MNS in our prior work [31]):

(1) Explainability: The “Stress + STIM” period in an experiment is classified as explainable with respect to the SMV (or a physio-marker) if the mean value of the SMV (or the physio-marker) is positive. The “STIM” period in an experiment is classified as explainable with respect to the SMV (or a physio-marker) if the mean value of the SMV (or the physio-marker) is negative. An experiment is classified as explainable with respect to the SMV (or a physio-marker) if both “Stress + STIM” and “STIM” periods therein are explainable with respect to the SMV (or the physio-marker). Hence, the explainability quantifies the degree to which the SMV (or a physio-marker) responds to acute mental stressors as well as MNS and AVNS in a physiologically explainable way.
(2) Stimulation consistency: For an explainable experiment, its stimulation consistency with respect to the SMV (or a physio-marker) is defined as the percentage of explainable data points pertaining to the SMV (or a physio-marker) in the “STIM” period of the experiment. Hence, the stimulation consistency quantifies the degree to which the SMV (or a physio-marker) response is maintained in the explainable (i.e., negative) direction.
(3) Stimulation sensitivity: For an explainable experiment, its stimulation sensitivity with respect to the SMV (or a physio-marker) is defined as the absolute mean value of the SMV (or the physio-marker) in the “STIM” period of the experiment. Hence, the stimulation sensitivity quantifies the degree to which MNS or AVNS can change the SMV (or a physio-marker).
(4) Stimulation effectiveness: For an explainable experiment, its stimulation effectiveness with respect to the SMV (or a physio-marker) is defined as the ratio between the absolute mean value of the SMV (or the physio-marker) in the “STIM” period of the experiment (i.e., the stimulation sensitivity) to the sum of the mean value of the SMV (or the physio-marker) in the “Stress-STIM” and “STIM” periods. Under the plausible assumption that the mean value of the SMV (or a physio-marker) in the “Stress-STIM” period is its acute stress-induced arousal minus its mitigation due to MNS or AVNS, and that the mitigation due to MNS or AVNS remains identical in both “Stress-STIM” and “STIM” periods, the stimulation effectiveness quantifies the degree to which MNS or AVNS can mitigate the acute stress-induced arousal pertaining to the SMV (or a physio-marker).

In calculating the above metrics pertaining to the periods of interest in the experiments (namely, “Stress + STIM” and “STIM” periods in Figure 1), we discarded the initial 30 s interval corresponding to the length of the smoothing window (see Section 2.2).

To comparatively investigate the efficacy of MNS and AVNS, we compared the above metrics pertaining to the SMV. To comparatively investigate the mechanism of action of MNS and AVNS, we compared the SMV weights and the above metrics pertaining to individual physio-markers.

## 3. Results

**Figure 3:**
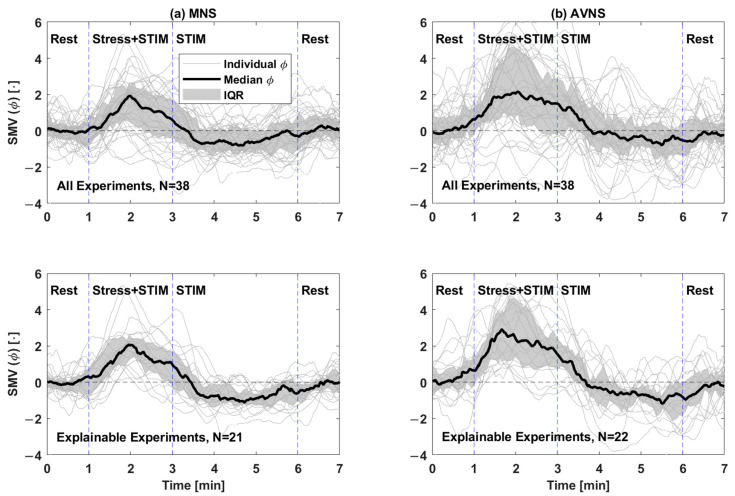
SMV responses to acute mental stress and stimulation pertaining to (**a**) MNS and (**b**) AVNS, both pertaining to all experiments (**upper panel**) and explainable experiments (**lower panel**). IQR: interquartile range. STIM: stimulation (MNS or AVNS).

**Figure 4:**
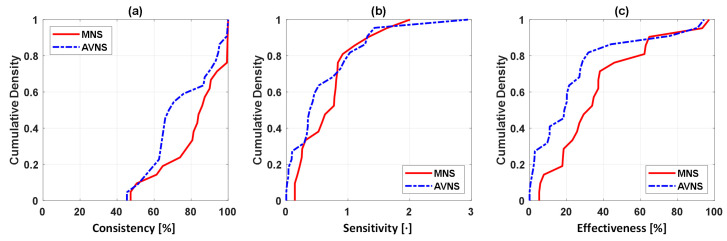
Stimulation consistency, stimulation sensitivity, and stimulation effectiveness between MNS and AVNS in terms of the SMV pertaining to the explainable experiments: (**a**) Cumulative density of stimulation consistency. Ideally, stimulation consistency must be 100% in all the experiments (i.e., the cumulative density of 1 at 100% and 0 otherwise). (**b**) Cumulative density of stimulation sensitivity. Ideally, stimulation sensitivity must be large. Hence, MNS is comparable or marginally superior to AVNS. (**c**) Cumulative density of stimulation effectiveness. Ideally, stimulation effectiveness must be large. Hence, MNS is superior to AVNS.

**Figure 5:**
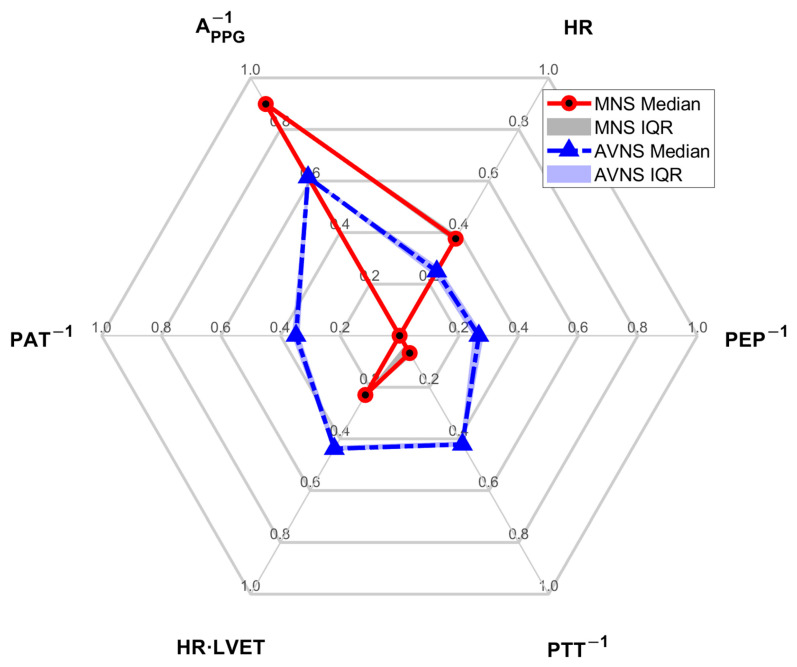
The weights of the 6 physio-markers composing the SMV pertaining to MNS and AVNS.

**Figure 6:**
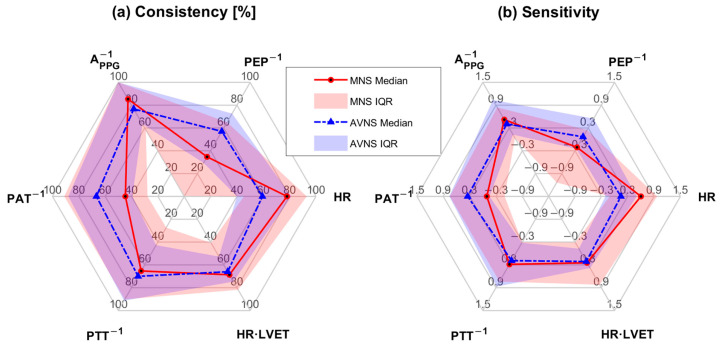
Stimulation consistency and stimulation sensitivity between MNS and AVNS in terms of the 6 physio-markers composing the SMV pertaining to the explainable experiments: (**a**) stimulation consistency [%]. (**b**) stimulation sensitivity.

Figure 3 shows the SMV responses to (a) acute mental stressor and MNS vs. (b) acute mental stressor and AVNS, both pertaining to all experiments (upper panel) and explainable experiments (lower panel). Table 1 compares the explainability between MNS and AVNS in terms of the SMV. Table 2 compares stimulation consistency, stimulation sensitivity, and stimulation effectiveness between MNS and AVNS in terms of the SMV pertaining to the explainable experiments, and Figure 4 shows the cumulative density of the same metrics. Figure 5 shows the weights of the six physio-markers composing the SMV pertaining to MNS and AVNS. Table 3 compares the explainability between MNS and AVNS in terms of the six physio-markers composing the SMV. Table 4 compares the stimulation consistency and stimulation sensitivity between MNS and AVNS pertaining to the explainable experiments in terms of the six physio-markers composing the SMV, and Figure 6 shows the same metrics.

## 4. Discussion

Despite its prevalence and substantial deterioration of both physiological and psychological health and disease, prior work aimed at mitigating acute mental stress-induced arousal is limited. Conventional pharmacological interventions may be effective in dealing with chronic stress but cannot provide just-in-time mitigation of acute mental stress-induced arousal. Being non-invasive, compatible with wearable and hearable form factors, and known to mitigate acute mental stress-related physiological responses, transcutaneous peripheral neuromodulation has the potential to enable the ubiquitous management of acute mental stress-induced arousal. However, there is a major knowledge gap regarding the relative efficacy of MNS and AVNS as well as the differences between them. Hence, we compared MNS and AVNS in the context of their ability and mechanism to mitigate acute mental stress-induced arousal.

### 4.1. MNS vs. AVNS: Comparable Efficacy in the Mitigation of Acute Stress-Induced Arousal

In terms of the SMV, MNS and AVNS were comparable in the number of explainable experiments (Table 1 and Figure 3). However, within the explainable experiments, MNS exhibited stimulation consistency, stimulation sensitivity, and stimulation effectiveness superior to AVNS (Table 2 and Figure 4). First, in terms of descriptive statistics, all three metrics were higher in MNS than in AVNS. In particular, the stimulation effectiveness pertaining to MNS was significantly higher than AVNS. Second, the IQR pertaining to the stimulation sensitivity was smaller in MNS than in AVNS, meaning that MNS may be associated with less inter-individual variability in stimulation efficacy than AVNS. All in all, MNS appeared to be superior to AVNS, with comparable explainability and superior efficacy in the explainable experiments. However, it is also noted that MNS and AVNS were both adequately efficacious as follows. In terms of the SMV, MNS and AVNS appeared to be efficacious in >70% of the experiments (“STIM” in Table 1). In >60% of the explainable experiments, the anticipated stimulation responses were observed in >80% of the period during which MNS was exerted and >65% of the period during which AVNS was exerted (Figure 4a). In addition, MNS and AVNS were estimated to mitigate acute mental stress arousal by 34% and 19% on average, which is non-trivial if not substantial (Table 2).

### 4.2. MNS vs. AVNS: Mitigation of Acute Stress-Induced Arousal via Distinct Physio-Markers

The composition of the SMV pertaining to MNS and AVNS was notably different (Figure 5), which may imply that MNS and AVNS mitigate acute stress-induced arousal in different ways. In the case of MNS, A_PPG_^−1^ and HR had dominant weights relative to the other physio-markers, whereas, in the case of AVNS, all the physio-markers had somewhat uniform weights. Our prior work showed that all six physio-markers responded well to acute mental stressors [34,36] (although in the current work, HR did not appear to respond well to acute mental stressors while MNS was exerted (Table 3)). Hence, the degree of uniformity in the weights pertaining to the six physio-markers associated with a stimulation modality may be largely attributed to (i) whether or not the stimulation modality is efficacious in modulating all the physio-markers and (ii) whether or not the efficacy remains persistent across experiments (or equivalently, across participants). First, the explainability of the stimulation response pertaining to the six physio-markers was more diverse in MNS than in AVNS (“STIM” in Table 3). The coefficients of variation pertaining to MNS and AVNS were 0.26 and 0.20, respectively. In particular, the explainability pertaining to PEP^−1^ and PAT^−1^ was markedly low, meaning that the efficacy of MNS varied largely across participants in terms of these two physio-markers. In contrast, the explainability pertaining to AVNS was quite comparable across all six physio-markers, meaning that AVNS may be associated with a more or less roughly equal degree of efficacy to the six physio-markers across experiments and participants. Hence, MNS appears to exhibit more inter-individual variability than AVNS, or in other words, the efficacy of MNS is less persistent across experiments and participants than the efficacy of AVNS. Second, even within the explainable experiments, the efficacy of MNS pertaining to the six physio-markers was relatively more diverse than AVNS. In particular, MNS was not efficacious in modulating PEP^−1^ and PAT^−1^ relative to the other physio-markers, as indicated by low stimulation consistency and stimulation sensitivity values pertaining to PEP^−1^ and PAT^−1^ (Table 4 and Figure 6). In contrast, the efficacy of AVNS pertaining to the six physio-markers was less variable than MNS, as indicated by the narrower ranges pertaining to both stimulation consistency and stimulation sensitivity (in terms of both median and IQR values). Hence, MNS appears to exhibit more variability in its efficacy to modulate all six physio-markers than AVNS.

A_PPG_^−1^ stood out in the composition of both MNS and AVNS. Considering that A_PPG_^−1^ exhibited high explainability (“STIM” in Table 3) as well as high stimulation consistency and stimulation sensitivity in the explainable experiments (Table 4 and Figure 6), A_PPG_^−1^ may likely be the best physio-marker (among the six physio-markers) that can track the effect of both MNS and AVNS. Interestingly, this finding aligns with a prior work on transcutaneous cervical VNS [48]. On the other hand, PEP^−1^ and PAT^−1^ pertaining to MNS were associated with negligible weights. As mentioned above, MNS cannot modulate PEP^−1^ and PAT^−1^ relative to the other physio-markers, and as well, PEP^−1^ and PAT^−1^ responses to MNS are associated with large inter-individual variability. Hence, AVNS may be superior to MNS in mitigating arousal involving the changes in PEP^−1^ and PAT^−1^.

Finally, it is noted that the primary goal of this work was to compare and contrast MNS and AVNS in the context of their efficacy in mitigating acute stress-induced arousal. Hence, we did not intend to scrutinize neurophysiological mechanisms underlying the effect of MNS and AVNS on the mitigation of acute stress arousal. However, the findings in this work may foster deeper mechanistic investigations of how MNS and AVNS modulate autonomic nervous system functions.

### 4.3. Potential of MNS in Wearable-Enabled Acute Stress Management

The results pertaining to the SMV weights (Figure 5) in the current work also provide a practical perspective: MNS may have an advantage over AVNS in terms of real-world implementation of non-invasive peripheral neuromodulation technology. Indeed, the SMV pertaining to MNS may be approximated by as few as two physio-markers, namely, A_PPG_^−1^ and HR, based on their weight dominance. On the other hand, the SMV pertaining to AVNS may not be approximated by a small number of physio-markers due to the comparable and non-negligible weights pertaining to all the physio-markers. Such an advantage pertaining to MNS, combined with the superiority of MNS to AVNS in efficacy (in terms of SMV), may motivate the development of acute stress-induced arousal management technology based on the MNS. For example, a wristwatch platform capable of measuring the PPG signal (from which A_PPG_^−1^ and HR can be readily derived) can be armed with non-invasive transcutaneous stimulation circuitry to realize an MNS-enabled ubiquitous acute stress management system. In this way, the findings from the current work may contribute to the development of novel technologies for monitoring and mitigation of acute stress-induced arousal.

### 4.4. MNS and AVNS: Weakness

The current work has an important weakness related to the absolute degree of efficacy pertaining to MNS and AVNS. In our experimental data, an explainable SMV response was observed in ≥79% of the acute mental stress-stimulation periods (“Stress + STIM” in Table 1) and >71% of the stimulation periods (“STIM” in Table 1). However, the proportion of unexplainable experiments (namely, those in which the anticipated SMV response was not observed) was not negligible (up to around 27% in MNS and 29% in AVNS; see Table 1). In addition, the proportion of explainable experiments pertaining to the simulation period was even smaller at the level of individual digital biomarkers (“STIM” in Table 3). This non-ideal efficacy of MNS and AVNS may be attributed to two limitations among others. First, it is possible that MNS and AVNS were not efficacious in modulating acute stress-induced arousal in all experiments and participants. Second, it is possible that the MNS and AVNS administered in the current work did not stimulate the target nerves properly. In our opinion, the former is related to the fundamental limitation of peripheral neuromodulation techniques in general, while the latter is related to the practical limitation of non-invasive and transcutaneous stimulation in particular (as opposed to invasive stimulation in which stimulation of target nerves is guaranteed). Regardless of the root cause, the administration of inefficacious peripheral neuromodulation can induce discomfort without providing any benefit. In this context, effort must be invested to equip MNS and AVNS (and more generally, peripheral neuromodulation in general) with the capability to determine (i) if MNS/AVNS is given properly to stimulate the target nerves and (ii) if MNS/AVNS is efficacious. Such a novel capability may help develop ubiquitous, efficacious, and comfortable acute stress arousal monitoring and mitigation technologies.

## 5. Conclusions

We showed that MNS and AVNS may both have the potential to develop ubiquitous just-in-time non-invasive peripheral neuromodulation technologies to manage acute stress-induced arousal in healthy subjects without neuropsychiatric disorder. We also showed that MNS may require relatively fewer physiological sensing requirements than AVNS, while AVNS may be more efficacious than MNS in mitigating more diverse arousal patterns in healthy subjects without a neuropsychiatric disorder. The efficacy of MNS and AVNS in attenuating acute stress-induced arousal in other populations remains unknown and must be investigated. Future work to reinforce the findings in diverse populations and translate these findings to the development of practical technologies may contribute to the advancement of mobile mental health.

## Figures and Tables

**Table 1:** Explainability between MNS and AVNS in terms of the SMV.

	MNS	AVNS
Stress + STIM [%]	79.0	84.2
STIM [%]	73.7	71.1
Stress + STIM & STIM [%]	55.3	57.9

**Table 2:** Stimulation consistency, stimulation sensitivity, and stimulation effectiveness between MNS and AVNS in terms of the SMV pertaining to the explainable experiments (median (IQR)). *: *p* < 0.05 (Wilcoxon rank sum test).

	MNS	AVNS
Consistency [%]	86.0 (76.5–99.5)	69.3 (63.3–93.3)
Sensitivity	0.77 (0.26–0.86)	0.40 (0.10–0.94)
Effectiveness [%]	33.8 (18.3–49.8) *	19.4 (2.90–28.8)

**Table 3:** Explainability between MNS and AVNS in terms of the 6 physio-markers composing the SMV.

	MNS		AVNS	
	Stress + STIM [%]	STIM [%]	Stress + STIM [%]	STIM [%]
PEP^−1^	81.6	31.6	71.1	44.7
HR	39.5	65.8	63.2	50.0
A_PPG_^−1^	76.3	63.2	81.6	71.1
PAT^−1^	76.3	39.5	79.0	47.4
HR·LVET	71.1	55.3	68.4	68.4
PTT^−1^	76.3	57.9	71.1	57.9

**Table 4:** Stimulation consistency and stimulation sensitivity between MNS and AVNS in terms of the 6 physio-markers composing the SMV pertaining to the explainable experiments (median (IQR)).

	MNS		AVNS	
	Consistency [%]	Sensitivity	Consistency [%]	Sensitivity
PEP^−1^	34.7 (0.50–65.0)	−0.21 (−1.13–0.25)	57.0 (29.3–72.7)	0.07 (−0.25–0.55)
HR	78.0 (45.3–93.0)	0.60 (−0.11–0.97)	59.3 (40.0–81.3)	0.15 (−0.21–0.50)
A_PPG_^−1^	85.3 (58.3–100)	0.52 (0.04–0.84)	76.7 (63.3–100)	0.41 (0.12–1.04)
PAT^−1^	44.7 (27.8–91.0)	−0.09 (−0.50–0.75)	66.7 (39.3–88.0)	0.34 (−0.15–0.76)
HR·LVET	68.7 (40.7–81.8)	0.25 (−0.29–0.82)	66.0 (52.7–74.7)	0.21 (−0.13–0.39)
PTT^−1^	65.3 (27.8–90.2)	0.29 (−0.29–0.74)	70.0 (42.7–91.3)	0.19 (−0.28–0.87)

## Data Availability

The raw data supporting the conclusions of this article will be made available by the authors on request.
